# Remotely monitored *Baduanjin* exercise in moderate-to-severe chronic obstructive pulmonary disease patients (BROCADE): A study protocol

**DOI:** 10.1097/MD.0000000000032079

**Published:** 2022-12-30

**Authors:** Shuanglan Xu, Zhifei Yin, Zi Chen, Dandan Zhang, Sheng Ye, Ping Zhou, Aiping Chen, Di Wu, Weihua Liu, Liuchao Zhang, Liquan Guo, Guangxu Xu, Linfu Zhou

**Affiliations:** a Department of Respiratory and Critical Care Medicine, The First Affiliated Hospital, Nanjing Medical University, Nanjing, China; b Department of Geriatric Rehabilitation Medicine, Centre of Rehabilitation Medicine, The First Affiliated Hospital, Nanjing Medical University, Nanjing, China; c Department of Respiratory Medicine, Geriatric Hospital of Nanjing Medical University, Nanjing, China; d Department of Respiratory Medicine, BenQ Hospital of Nanjing Medical University, Nanjing, China; e School of Biological Sciences and Medical Engineering, Southeast University, Nanjing, China; f Department of Radiology, The First Affiliated Hospital, Nanjing Medical University, Nanjing, China; g Suzhou Institute of Biomedical Engineering and Technology, Chinese Academy of Sciences, Suzhou, China; h Institute of Integrative Medicine, Nanjing Medical University, Nanjing, China.

**Keywords:** *Baduanjin*, chronic obstructive pulmonary disease, moderate-to-severe, randomized controlled trial, remote monitoring

## Abstract

*Baduanjin* is a traditional Chinese exercise regimen used to treat various chronic illnesses and is associated with both psychological and physical benefits. However, its benefits for patients suffering from chronic obstructive pulmonary disease (COPD) are unclear. This study aims to assess the efficacy, safety, and underlying mechanisms of *Baduanjin* exercise in patients with moderate-to-severe COPD (BROCADE) by remote monitoring.

**Methods:**

This study protocol describes a multicenter, open-label, prospective randomized computed tomography. A total of 150 individuals who meet the inclusion criteria after the screening and consent processes will take part in the study. All participants will be provided routine medication and lifestyle interventions. They will be randomly assigned to a control group, a classical pulmonary rehabilitation group, or a *Baduanjin* group, which will undergo remotely monitored *Baduanjin* exercises for a cumulative duration of 1 hour per day, three times per week for 12 weeks. The participants will be followed for 24 weeks. The primary outcomes will be a 6-minutes walking distance and St. George’s Respiratory Questionnaire index. The secondary outcomes will be lung function, cross-sectional area of the pectoralis major and subcutaneous fat, modified Medical Research Council score, COPD assessment test questionnaire results, extremity muscle strength, and quality of life. Any adverse events that may occur will be monitored and recorded.

**Results:**

This study is ongoing and will be submitted to a peer-reviewed journal for publication once completed.

**Conclusion:**

A novel neutrophil-related inflammatory mechanism will potentially be identified. In addition, the study results will provide a safe, effective, simple and operational *Baduanjin* exercise protocol for moderate-to-severe COPD patients aimed at improving prognosis and quality of life.

## 1. Introduction

Globally, chronic obstructive pulmonary disease (COPD) is a disabling and progressive lung disease with recurrent symptomatic dyspnea, resulting in a high rate of re-hospitalization, mortality, and disability. It is anticipated that by 2030, COPD will be the third leading cause of death worldwide, resulting in a heavy social and economic burden.^[1–3]^ When treating COPD, it is important to focus on improvement of symptoms, reduction of the frequency and severity of exacerbations, and improvement in exercise tolerance and health status.^[4]^ However, there are no specific treatments for preventing and reversing the decline in lung function. Therefore, effective new approaches are urgently needed to improve the prognosis and quality of life of patients with COPD.

With the rapid development of the Internet and advanced technology, telemedicine and telecare models have been adopted globally as new medical services beneficial for earlier diagnosis, treatment, and improved self-management in chronic diseases.^[5]^ Telemedicine may be a suitable and crucial method for the delivery of remote education or other interventions in China because of its large population and shortage of medical resources.^[6]^ Compared with the usual home-based model, telemedicine and telecare models provide new alternatives to hospital-based care, with a virtual ward that can monitor respiratory function and improve the management of COPD, including early detection of acute exacerbations and improved health-related outcomes.^[7]^ Furthermore, post-discharge telemonitoring services could decrease COPD-related hospital re-admissions and mortality rates derived from acute exacerbation.^[8,9]^ Since December 2019, the global number of cases of coronavirus disease 2019 (COVID-19) has been rapidly increasing. Given the importance of avoiding person-to-person transmission during the COVID-19 pandemic, remote home monitoring combined with rehabilitation treatment may help keep patients with COPD or other chronic diseases safe, and this has been recognized by the Global Initiative for Chronic Obstructive Lung Disease (GOLD) guidelines.^[10]^

Pulmonary rehabilitation (PR) is an important non-pharmacological treatment that significantly improves symptoms, quality of life, and exercise capacity in patients with COPD. Of note, exercise training has been recognized as one of the core interventions.^[11]^ *Baduanjin* Qigong is a traditional Chinese exercise regimen involving relaxation and slow movements, without the limitations derived from equipment or venue availability. *Baduanjin* exercise or other subtypes of Qigong have both psychological and physical benefits in patients with various chronic illnesses, including cardiovascular disease,^[12]^ musculoskeletal pain,^[13]^ and cancer.^[14]^ Although many studies have reported on the application of *Baduanjin* in patients with COPD,^[15]^ its efficacy, safety, and mechanism of action as a complementary exercise therapy have not been evaluated to this date.

A recent meta-analysis concluded that *Baduanjin* exercise could improve exercise capacity, lung function, and quality of life in COPD patients,^[16,17]^ but the assessment criteria were not comprehensive, the potential mechanisms of action were not investigated, and there was no valid comparison of *Baduanjin* with traditional PR. Therefore, the aim of this study is to evaluate the efficacy and safety of *Baduanjin* in patients with moderate-to-severe COPD (BROCADE) in a multicenter randomized controlled trial (RCT) setting under home remote monitoring, and to explore the underlying neutrophil-related inflammatory mechanism. Novel mechanisms and evaluation methods will be determined.

## 2. Methods

### 2.1. Design and registration

This will be a pilot multicenter, open-label, prospective, randomized controlled clinical trial aiming to evaluate the efficacy and safety of *Baduanjin* in patients with moderate-to-severe COPD under remote monitoring and to explore the possible mechanisms involved in remission of the inflammation. After qualified recruitment, screening, and random assignment, a total of 150 participants from the Department of Respiratory Medicine of three teaching hospitals in Nanjing (China) will be included. All participants will be provided routine medication and lifestyle intervention, and they will be randomly assigned to one of three groups: a control group that will receive education on *Baduanjin* exercise, a classical PR group, and a *Baduanjin* group that will undergo a remotely monitored *Baduanjin* exercise program. The primary and secondary outcomes will be measured at baseline and at the cutoff point every 4 weeks. The exercise will be conducted at the outpatient clinic and monitored by a smartphone-based remote rehabilitation software program. The exercise program will last for 12 weeks, and the patients will be followed up for 24 weeks.

**Figure 1.**
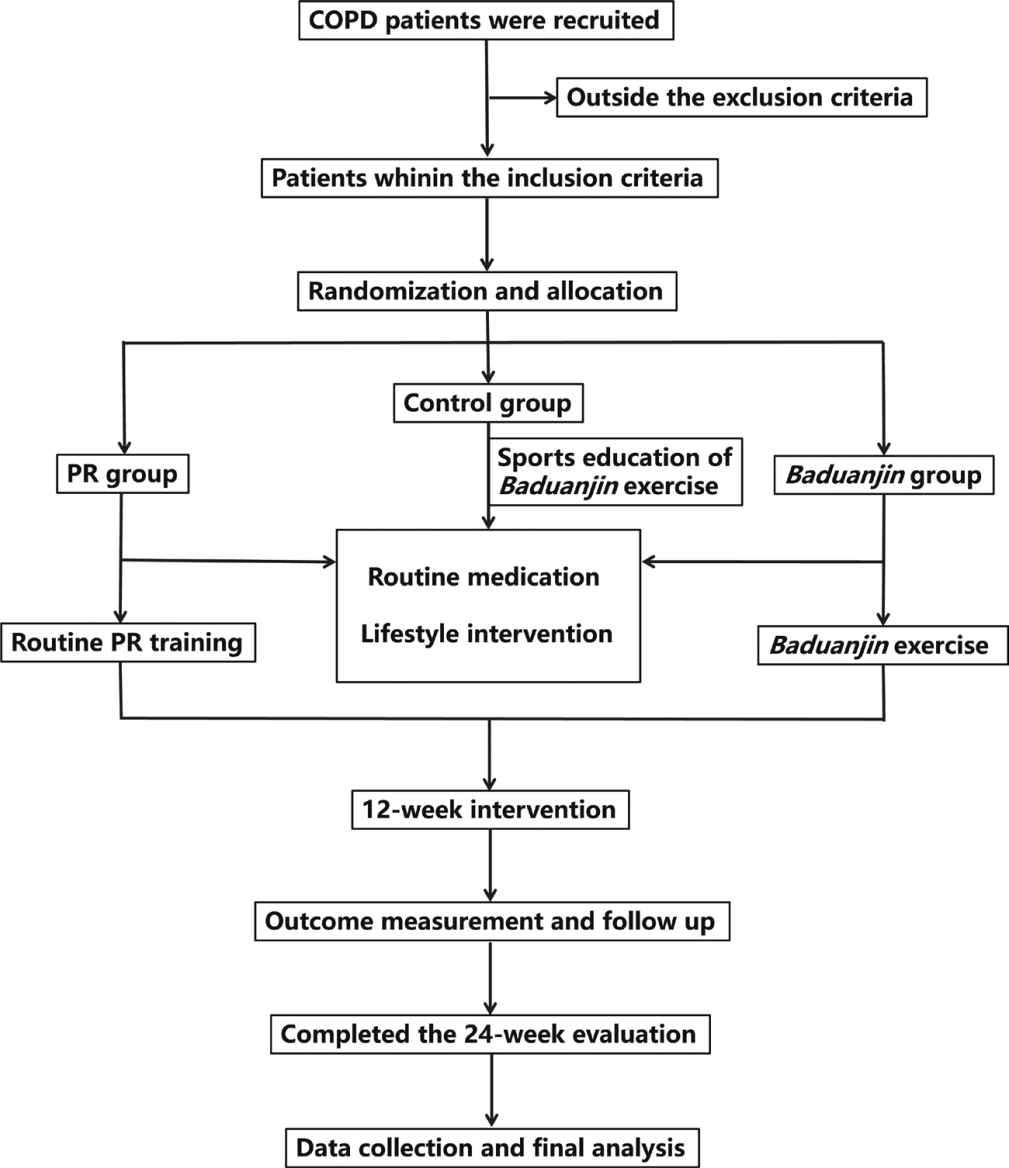
Flowchart of the study design. COPD = chronic obstructive pulmonary disease, PR = pulmonary rehabilitation.

**Figure 2.**
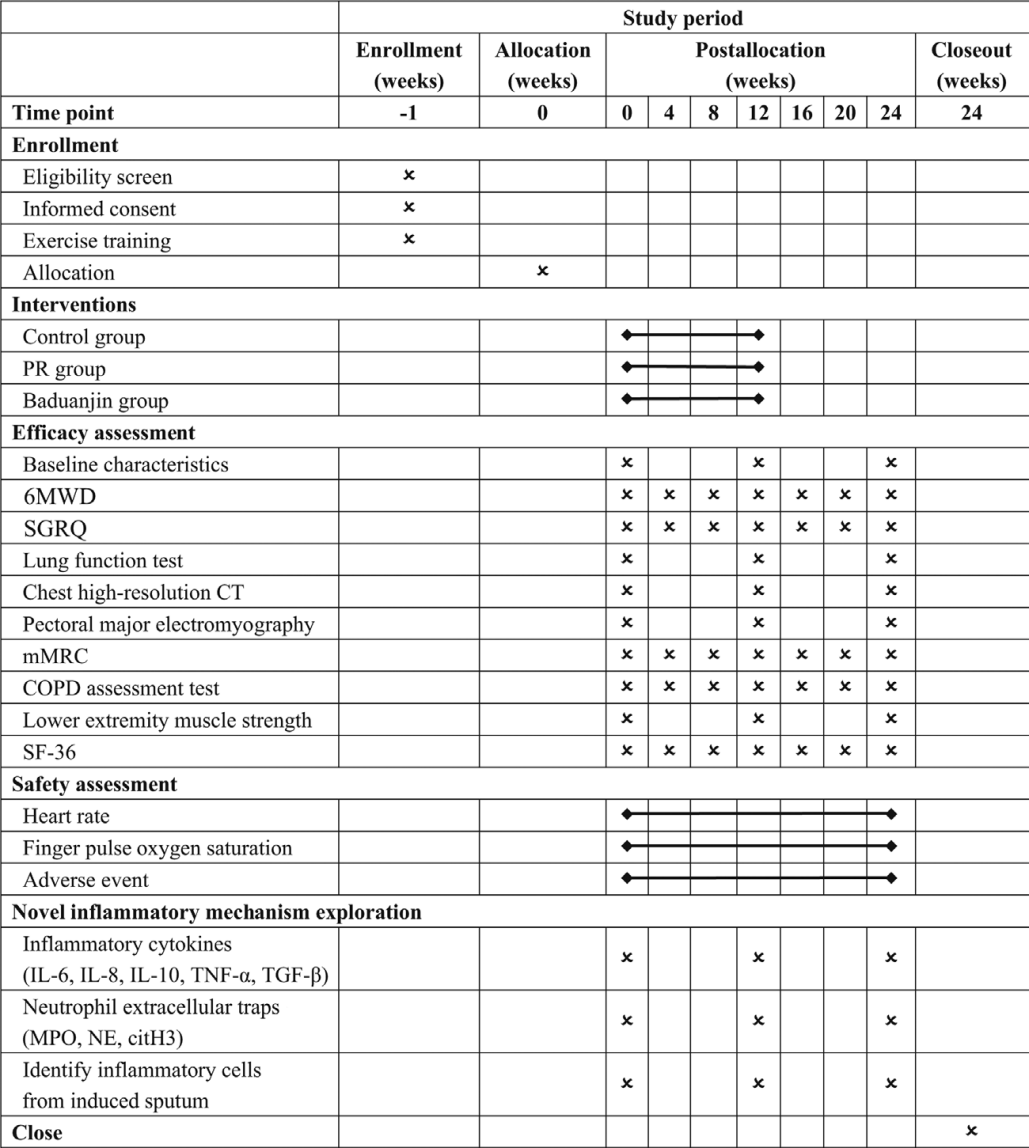
SPIRIT chart of the time points. 6MWD = 6-min walking distance, COPD = chronic obstructive pulmonary disease, CT = computed tomography, IL = interleukin, MPO = myeloperoxidase, NE = neutrophil elastase, PR = Pulmonary rehabilitation, TGF-β = transforming growth factor β, TNF-α = tumor necrosis factor α.

This work follows the Standard Protocol Items: Recommendations for Intervention Trials (SPIRIT).^[18]^ The flow chart of the participation screening process is shown in Figure 1, and the time points for enrollment, interventions, and assessments are shown in Figure 2. The study protocol has been registered with the Chinese Clinical Trial Registry (ChiCTR2100042700, http://www.chictr.org.cn/showproj.aspx?proj=120859).

### 2.2. Sample size calculation

As documented previously, the primary outcome of the current study will be the 6-min walking distance (6MWD) at 12 weeks. According to a literature review, traditional Chinese exercise regimen was reported to be superior to the control intervention in the improvement of cardiopulmonary function when assessed using the 6MWD (580 ± 52.5 m in the *Tai Chi* group vs 564 ± 68 m in the control group, *P *= .035).^[19]^ In addition, significant improvement was observed in the PR group as compared to the control group (472 ± 62.56 m in the PR group vs. 355.49 ± 56.72 m in the control group, *P *< .001). We hypothesized that *Baduanjin* and PR would be superior to the control intervention in the improvement of cardiopulmonary function. Therefore, a sample of 40 to 43 patients (based on the estimation of data from Polkey et al^[19]^) could be assumed to have 80% power to detect significant differences between groups (*P* = .05, two-sided), with 20% inflation to account for dropouts. As a three-arm designed RCT, we adopted 50 patients per center (a final sample size of 150) to power the effect size of pre-specified interventions. The Power Analysis and Sample Size (PASS) software (v. 15.0.5; NCSS, LLC, Kaysville, UT) will be used in this study.

### 2.3. Participant recruitment strategy

Individuals were recruited via poster or WeChat invitation in collaboration with three participating centers from January 2021 to June 2021. Additionally, potential participants were recruited using clinical databases at the three teaching hospitals by phone or face-to-face interviews. After being provided with information about the study, individuals who agree to participate will be asked to attend the screening evaluation.

### 2.4. Inclusion criteria

The inclusion criteria are a diagnosis of clinically stable COPD, and severity of airflow limitation classified as moderate-to-severe (30% ≤ forced expiratory volume in 1 s, [FEV_1_]_ _< 80% predicted value) based on post-bronchodilator FEV_1_, according to the GOLD criteria^[20]^; age of 40 to 75 years; ability to complete exercise training; ownership of a smartphone and ability to use it; no participation in other clinical trials in the past 6 months; no current participation in daily exercise, such as *Baduanjin*, *Tai Chi*, and aerobic exercises; and willingness to participate in research and sign the written informed consent form.

### 2.5. Exclusion criteria

The exclusion criteria are mild COPD (FEV_1 _≥ 80% predicted value) or very severe COPD (FEV_1 _< 30% predicted value); coexisting airway obstruction caused by asthma or other non-chronic obstructive pulmonary diseases; severe comorbidities, such as cardiovascular, kidney, or liver disease, malignant tumors, hematopoietic diseases, endocrine diseases, or mental illness; uncontrolled chronic diseases, including hypertension (resting blood pressure ≥ 160/100 mm Hg), diabetes (random blood glucose > 16.7 mmol/L, glycosylated hemoglobin > 7.0%); patients who are breastfeeding or pregnant or plan to become pregnant during the study period; lack of general athletic ability, including the upper and lower limbs; poor compliance, difficulty in cooperating with assessment, training, and treatment; and participation in any PR program within 4 weeks prior to enrollment or plan to participate in other PR programs during the study period.

### 2.6. Randomization

Statisticians who will not participate in the study will use the SPSS software (v. 24.0; IBM Corp., Chicago, IL) to generate random numbers, which will be placed in lightproof envelopes. Eligible participants will be assigned an envelope with group information.

### 2.7. Interventions

**Figure 3.**
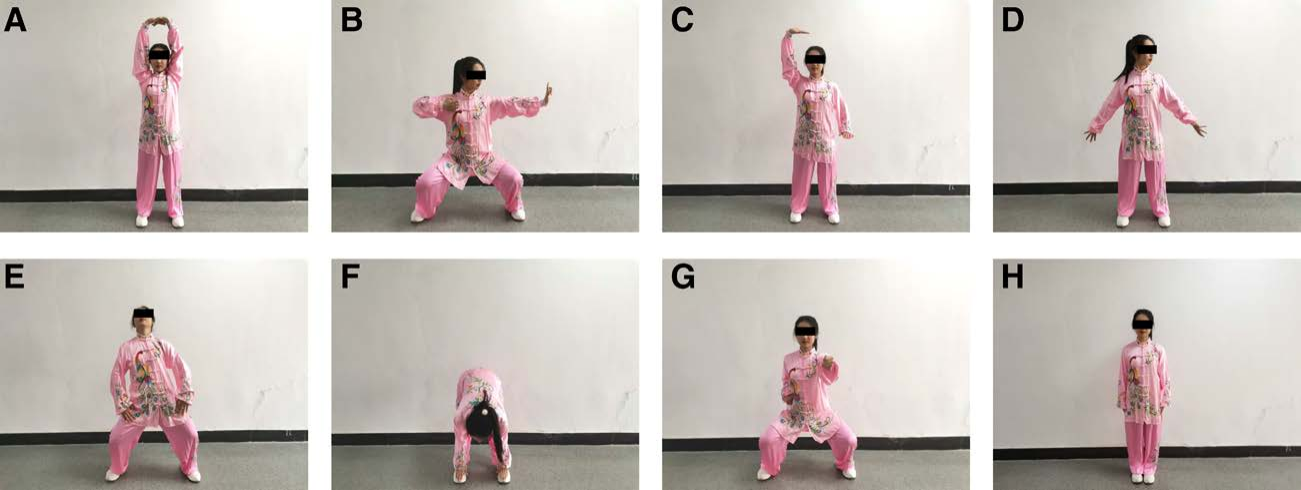
Eight forms of *Badunjin* Qigong exercise.

All participants will be given routine medication and lifestyle intervention, and they will be randomly assigned to one of three groups: a control group, which will receive health education involving exercise methods, health regimens, and nutrition The participants will receive a health education brochure and will be followed up by phone call to discuss physical activity, disease progression, health status, and psychological status; a classical PR group, which will undergo PR training in the outpatient clinic, including power bicycle training, upper limb resistance training, and lower limb resistance training; and a *Baduanjin* group, which will perform *Baduanjin* exercise, displayed by video instructions on the participants’ smartphones installed with a remote rehabilitation software (Sukang R + health App, v. 5.1.40.0, Chengdu Shangyi Information Technology Co., Ltd., Chengdu, China) whilst wearing a heart rate belt (RecoveryPlus H1, Chengdu Shangyi Information Technology Co., Ltd.), which will be monitored remotely. Before the start of the experiment, professionals from Nanjing University of Traditional Chinese Medicine will teach the *Baduanjin* exercise (version from the General Administration of Sports) until the participants master the correct posture and stretching. Eight forms of *Baduanjin* Qigong exercise are shown in Figure 3. In both the PR and *Baduanjin* groups, the exercise intervention regimen will need to be performed for a cumulative time of 1 h per day, three times per week for 12 weeks.

### 2.8. Efficacy assessments

The effects of *Baduanjin* exercise, including clinical symptoms, quality of life, activity and endurance, lung function, and respiratory muscle function, will be evaluated in patients with moderate-to-severe COPD and compared with the effects of the control and PR interventions. The primary outcomes will include the 6MWD and the St. George’s Respiratory Questionnaire index. The secondary outcomes will include lung function test results, including FEV_1_, forced volume capacity (FVC), ratio of FEV_1_ to FVC (FEV_1_/FVC%), maximum expiratory flow rate, and maximum ventilation per minute; chest high-resolution computed tomography to compare the cross-sectional area of the pectoralis major and subcutaneous fat; Modified Medical Research Council scale; COPD assessment test questionnaire results; lower extremity muscle strength; and Short-Form 36-item Health Survey for healthy living results.

### 2.9. Safety assessments

Although *Baduanjin* exercise is non-strenuous, easy to learn and practice, and unlikely to cause injury,^[21]^ we will evaluate its safety. During the process of exercise intervention, we will monitor the heart rate, finger pulse oxygen saturation, and adverse events (such as dyspnea, dizziness, falls, muscle strain, or COPD exacerbation) of each participant in real time. If any of the above events occur, the training will be stopped, and we will provide professional medical care, emergency observation, or hospitalization in severe cases. These events will be treated as reasons for automatic withdrawal from our study to comply with ethical rules. The details of adverse events will be recorded in the case report file and will be reported to the Ethics Committee within 24 hours.

### 2.10. Exploration of novel inflammatory mechanism

To characterize the inflammatory process in the participants, and the impact of the exercise program on it, 20 mL of elbow venous blood will be centrifuged and the serum will be used to detect common inflammatory cytokines by enzyme-linked immunosorbent assay, including interleukin (IL)-6, IL-8, IL-10, tumor necrosis factor-α, and transforming growth factor-β. Previous studies have confirmed that neutrophils play a pivotal role in the development and progression of COPD; however, the underlying mechanism remains unclear. Therefore, we will collect induced sputum from the participants to identify inflammatory cells by cell smear and staining techniques, including myeloperoxidase, neutrophil elastase, and histone citrullination of neutrophil extracellular traps (NETs) by enzyme-linked immunosorbent assay or western blotting. The expression of sialic acid-binding immunoglobulin-like lectin 9 (Siglec-9) will be tested by western blotting of samples obtained from the peripheral blood of the participants.

### 2.11. Data management

The statistician will randomize subjects into different groups and perform the statistical analyses. The Clinical Practice Center of three teaching hospitals will be responsible for monitoring research progress and securely storing the data and records. Only authorized research assistants will have access to the trial data.

### 2.12. Statistical analysis

Statistical software (SPSS version 24.0, SPSS, IL) will be used for statistical analysis in this study. All data will be presented as descriptive row statistical analyses (mean ± standard deviation). For comparisons between the three groups, analysis of variance will be used in cases in which the normal distribution and homogeneity of variance assumptions have been verified; otherwise, the Wilcoxon rank sum test will be used. For intra-group comparisons, an independent sample *t* test will be used in cases in which the normal distribution and homogeneity of variance assumptions have been verified; otherwise, a paired sign rank sum test will be used. For baseline unequal data, the two groups will be compared by covariance analysis. Additionally, statistical data will be used to calculate the composition ratio and the rate of each indicator. The total effective rate will be compared between the groups using the c*hi*-squared test. Statistical significance will be set at *P* < .05.

### 2.13. Patient and public involvement

Based on patients’ priorities, experiences, and preferences, professionally trained staff will objectively evaluate the development of the research question, outcome measures, and burden of each intervention, and this process will be supervised by another staff member. Additionally, individual data will not be reported back to the participants. None of the recruited participants has been involved in the study design, drafting of the manuscript, data collection, analysis, and interpretation, nor in any decision related to the publication of the results.

### 2.14. Ethics and dissemination

Before the participants provide written informed consent, the researcher will be responsible for comprehensively introducing the purpose, procedures, and possible benefits and risks. The study protocol has been approved by the Ethics Committee of the First Affiliated Hospital of Nanjing Medical University (No. 2021-SR-024), Geriatric Hospital of Nanjing Medical University (No. 2021-010), and BenQ Hospital of Nanjing Medical University (No. 2021-KL011). Written informed consent will be obtained from all participants. Study results will be disseminated through peer-reviewed publications and academic conferences.

## 3. Discussion

The purpose of this study will be to evaluate the efficacy, safety, and potential mechanism of *Baduanjin* exercise under remote monitoring in patients with moderate-to-severe COPD. In the BROCADE study, a *Baduanjin* group will be compared to a control group and a PR group, to determine whether or not *Baduanjin* exercise has positive and significant effects on lung function, exercise capacity, cross-sectional area of the pectoralis major and subcutaneous fat, health status, mental status, and quality of life in patients with moderate-to-severe COPD. If this trial is successful, we will be able to conclude that *Baduanjin* exercise can help improve prognosis and quality of life, and provide a promising and effective adjuvant therapy program for moderate-to-severe COPD.

COPD is a complex multisystem disease that can lead to impaired lung function and some systemic manifestations, such as peripheral muscle dysfunction or wasting, cachexia, and negative effects on physiology and psychology. Eventually, this disorder can affect daily activities and lead to disability and premature mortality.^[22]^ Although current therapies may temporarily improve symptoms, they have limited effectiveness in preventing the progressive decline of lung function, the progression of the disease, and the frequency of acute exacerbation. There is an urgent need to determine precise treatments. Based on the GOLD categories,^[20]^ COPD severity is classified as mild (FEV_1 _≥ 80% predicted value), moderate (50% ≤ FEV_1 _< 80% predicted value), severe (30% ≤ FEV_1 _< 50% predicted value), and extremely severe (FEV_1 _< 30% predicted value) in clinical practice. Reports suggest that the outcome of exercise PR in mild COPD patients may not be obvious, and extremely severe COPD patients are susceptible to adverse events. Therefore, moderate-to-severe (30% ≤ FEV_1_ < 80% predicted value) COPD patients with athletic ability will be enrolled in our study to explore the efficacy and safety of treatment.

Computed tomography (CT) is the gold standard for morphological evaluation of lung tissue in vivo, and the imaging phenotype of CT is able to quantify morphological changes of emphysema, large airway obstruction, and air trapping in COPD patients.^[23,24]^ Recently, a study concluded that the CT phenotype of airway surface area-to-volume ratio is an imaging biomarker of airway remodeling, which could provide differential information on predominant airway narrowing and loss and reveal respiratory morbidity, decline of lung function, and survival in COPD.^[25]^ Furthermore, other novel values of the CT phenotype in COPD and other lung diseases need to be explored. In our study, the pectoralis major, a breathing-aid muscle, will be assessed as a promising new evaluation indicator of COPD rehabilitation by high-resolution computed tomography based on the algorithm we developed, compared to the cross-sectional area of the pectoralis major and subcutaneous fat. This is a highlight of the present study. Previous studies concluded that loss of skeletal muscle mass or thickness changes are associated with poor prognosis and disease severity in COPD.^[26–29]^ There is no doubt that the mechanisms by which the condition of the pectoralis major contributes to COPD PR will be revealed in the future.

Chronic airway inflammation is identified as a key factor in the pathogenesis of COPD, characterized by an increase in inflammatory cytokines, including IL-6, IL-8, IL-10, tumor necrosis factor-α, and transforming growth factor-β, which play an important role in the occurrence and development of COPD.^[30]^ Of note, neutrophilic inflammation predominates in the COPD airway lumen and wall, and it can even predict the process and outcomes of this disorder.^[31,32]^ Neutrophils are one of the host’s defense lines against pathogen invasion and they act as phagocytes to remove pathogens with the assistance of cytokines and chemotactic agents. Moreover, neutrophils form reticular ultrastructures known as NETs and release various proteins outside of the cell by degranulation, including myeloperoxidase, neutrophil elastase, and Cit-H3.^[33]^ Compared with healthy controls, accumulation of NETs is significantly increased in COPD sputum and serum, and high NET concentrations are positively correlated with the severity and exacerbation of COPD.^[34]^ However, the role of NETs in COPD pathogenesis remains unclear. Focusing on targeted NETs may be a potential treatment strategy for COPD.

Additionally, much attention has been focused on Siglec-9, which is mainly expressed by human neutrophils and monocytes. Zeng et al^[35]^ confirmed that the expression of Siglec-9 was elevated in various tissues, including plasma, resulting in increased production of human alveolar and peripheral blood neutrophils in COPD patients, which is increased in vitro from peripheral blood neutrophils and culture supernatant by lipopolysaccharides, cigarette smoke extract, dexamethasone, and some cytokines.The polymorphism of Siglec-9 is closely related to the COPD exacerbation-prone phenotype, and the Siglec-9 variant rs2075803 G/rs2258983 A haplotype could be a risk factor for the development of emphysema.^[36]^ Interestingly, Siglec-9 binding with specific anti-Siglec-9 monoclonal antibodies or natural anti-Siglec-9 autoantibodies in human intravenous immunoglobulin (IVIg) preparations can induce neutrophil death by apoptosis, and generate reactive oxygen species involved in cell death signals.^[37,38]^ In our recent study, we hypothesized that an efficient treatment against neutrophil-associated inflammation could be developed using Siglec-9 ligands or agonistic antibodies, and Siglec-9 may be a potential therapeutic target for COPD.^[39]^ Therefore, in this study, we will collect samples of the induced sputum and peripheral venous blood of participants to identify inflammatory cells, inflammatory cytokines, and the expression of NETs and Siglec-9 using a variety of molecular biology methods. Furthermore, the presence of neutrophil-related NETs and Siglec-9 inflammatory mechanisms will be investigated in vitro and in vivo in the future.

*Baduanjin* Qigong has been a part of healthcare for thousands of years in China as part of the theory of traditional Chinese medicine, and it involves eight sections of simple and easy-to-learn movements to smooth Qi and blood and regulate the Yin-Yang balance.^[40]^ Previous studies have confirmed that *Baduanjin* is an aerobic exercise that improves respiration by increasing gaseous exchange and vital capacity.^[41]^ Additionally, this exercise could reduce fatigue, anxiety, and depression symptoms in people with physical or mental illnesses, as well as improve sleep quality and muscle strength of the spine and extremities.^[42,43]^ *Baduanjin* intervention showed a significant improvement in the 6MWD results of patients with mild-to-moderate Parkinson’s disease^[44]^; hence, it may be an effective measure for improving limb function. Notably, a recent meta-analysis concluded that *Baduanjin* exercise could improve lung function, exercise capacity, and quality of life in patients with COPD,^[16,17]^ but there is a lack of evidence from high-quality clinical trials to confirm these findings. Therefore, it is reasonable to expect that the *Baduanjin* exercise is suitable for patients with moderate-to-severe COPD who have low exercise tolerance.

Several strengths are associated with our trial, the BROCADE study. First, the results will be reliable and convincing given the objective measurement of the primary and secondary outcomes. Second, we will combine remote monitoring with smart mobile phone rehabilitation software and monitor the participants’ exercise status and the data in real time.^[45]^ At the same time, we will also set up an alarm system for abnormal heart rate to ensure safety. The device has already been successfully tested. Once it is widely adopted, this device may reduce the social and medical burden of COPD. However, a limitation of this study is that exercise coaches cannot be blinded; thus, the assessment results might be affected by subjective bias. Additionally, the generalizability is limited by the fact that the patients come from a single geographical area. Increased efforts will be needed to carry out multicenter RCTs and multi-dimensional comprehensive comparisons in the future.

In summary, this multicenter RCT will evaluate the efficacy, safety, and potential neutrophil-related inflammatory mechanism of *Baduanjin* exercise by remote monitoring of patients with moderate-to-severe COPD. The BROCADE study is ongoing. We hope that the findings of this trial will show that *Baduanjin*, a safe, effective, simple, and operational exercise, is beneficial for the prognosis and quality of life in patients with moderate-to-severe COPD.

## Acknowledgments

The authors are grateful to all the participants for their advice and support for the BROCADE study. We also thank Minlan Chen and Suzhou Changfeng Pharmatech Inc., for technical assistance in *Baduanjin* exercise under remote monitoring.

## Author contributions

**Conceptualization:** Linfu Zhou.

**Data curation:** Zhifei Yin, Zi Chen, Dandan Zhang, Sheng Ye, Liquan Guo, Guangxu Xu, Linfu Zhou.

**Funding acquisition:** Linfu Zhou.

**Investigation:** Shuanglan Xu, Zi Chen, Dandan Zhang, Sheng Ye, Di Wu, Weihua Liu, Liuchao Zhang, Linfu Zhou.

**Methodology:** Zhifei Yin, Linfu Zhou.

**Project administration:** Linfu Zhou.

**Resources:** Aiping Chen.

**Software:** Ping Zhou, Aiping Chen, Liquan Guo.

**Supervision:** Guangxu Xu, Linfu Zhou.

**Writing – original draft:** Shuanglan Xu.

**Writing – review & editing:** Liquan Guo, Guangxu Xu, Linfu Zhou.

## Corrections

“This work was supported by grants from the National Key Research and Development Program of China (Grant No. 2022YFF0710800), in the first page.” was removed from the funding information.
